# Influence of Noble Metals on Morphology and Topology of Structural Elements in Magnesium Alloy

**DOI:** 10.3390/ma17174173

**Published:** 2024-08-23

**Authors:** Viktor L. Greshta, Vadim A. Shalomeev, Oleksandr S. Lukianenko, Rafał Bogucki, Kinga Korniejenko, Serhii S. Tabunshchyk

**Affiliations:** 1 National University Zaporizhzhya Polytechnik, 64 Zhukovs’kogo Street, 69061 Zaporizhzhia, Ukraine; greshtaviktor@gmail.com (V.L.G.); shalomeev@zp.edu.ua (V.A.S.); saneklukyanenko@gmail.com (O.S.L.); 2 Faculty of Materials Engineering and Physics, Cracow University of Technology, Warszawska 24, 31-155 Cracow, Poland; rafal.bogucki@pk.edu.pl

**Keywords:** magnesium alloy, noble metals, microstructure, intermetallics, mechanical properties, implant

## Abstract

The main motivation for this study was to improve implant materials. The influence of silver and gold on the structure and mechanical properties of Mg–Nd–Zr alloy was studied. In the work, quantitative and qualitative evaluation of the structural components of magnesium alloy with noble metal additives was performed. The research methods used were investigation of the mechanical properties and observation of micro– and macrostructures. The results showed that modification of magnesium alloy with Ag and Au contributes to the formation of spherical intermetallics of smaller size groups, which become additional centers of crystallization and grind the cast structure. The best composition from additional alloying with silver and gold was determined. Their positive effect on the strength and ductility properties of the metal was established. Preclinical and clinical testing was performed and the prospects for noble metal modification of bioabsorbable magnesium alloy for implant production usage were shown.

## 1. Introduction

In contemporary medicine, titanium alloy and stainless-steel implants are extensively employed for osteosynthesis procedures. However, during intensive bone regeneration, they slow down bone growth and are not effective [1,2]. Inflammatory processes could also develop around them, leading to further bone loosening [3]. In addition, such implants must be removed to stabilize fractures after the bone fragments have fused, which requires a second surgery. To eliminate this drawback, bioresorbable materials are used [4,5]. The most common among these are various polyacids, the usage of which eliminates the removal operation. However, due to the inferior mechanical properties of polyacids, it is complicated to use them for osteosynthesis [6].

Magnesium could be a promising biosoluble material for implants [7,8]. Its positive effect on the human body is known. Magnesium is a biologically compatible material because the adult human body contains about 140 g of magnesium (0.2% of body weight), with 2/3 of this amount being in bone tissue; the daily magnesium requirement for an adult is 400 to 500 mg. Its biodegradation products are well absorbed [9,10]. This element optimally matches skeleton properties, ensures good fusion of bone fragments and quickly restores them at the site of a fracture [11,12]. However, pure magnesium metal provides neither the required levels of mechanical properties nor the biosolubility of osteosynthesis structures made of it. A solution to this problem can be the use of magnesium-based alloys that have significantly better mechanical properties than pure metallic magnesium [13,14]. Therefore, most studies are devoted to improving the physical, mechanical, and special properties of magnesium alloys by improving the composition of existing alloys and creating new ones [14,15]. At the same time, the chemical composition of magnesium alloys plays a significant role in the formation of the structures and properties of magnesium alloys [16,17]. Combined with smelting and refining, alloying and modification determine the nature of crystallization, the degree of granularity of the structure, and the set of alloy properties [18]. However, until now, the relationship between the morphology and topology of structural components during the modification of magnesium alloys and the resulting mechanical properties has not yet been established.

The primary stage of melt solidification, associated with the appearance of crystallization germs, is the basis for the formation of the structure and properties of the alloy. Therefore, the process of forming a solid metal from a melt is determined by modifying elements that can form additional crystallization centers. When smelting various alloys, elements that can react with the chemical components of the alloy and form refractory particles are widely used as modifiers. These modifiers form a highly dispersed system in the melt, which leads to a significant increase in crystallization centers, changes the morphology and topology of the alloy’s structural components in its solid state, and improves its mechanical properties. At the same time, when selecting modifying elements, it is necessary to take into account a number of operational requirements that are determined by the specifics of the use of the final products.

For example, one of the main requirements for modifiers of medical magnesium alloys is their non-toxicity. Silver and gold, which have long been used by mankind in everyday life and medicine, could be promising modifiers of magnesium alloys used for the manufacture of implants [19,20,21]. These elements form solid solutions with magnesium with limited solubility and are capable of intermetallic hardening. Intermetallic phases formed with magnesium can be released during the initial period of alloy crystallization and serve as additional crystallization centers, crushing the grain and positively affecting its properties [8].

Therefore, it is of practical interest to study the effect of Au and Ag on the structure formation and mechanical properties of magnesium alloys used for the manufacture of implants in osteosynthesis. We investigated separate and joint effects of silver and gold in amounts of 0.05–0.2% each on the morphology and topology of the formed intermetallic phase and their influence on the mechanical properties of NZ30K alloy, which is the most widely used in biological and medical research [22,23].

## 2. Materials and Methods

NZ30K alloy has the following chemical composition (according to DSTU ISO 16220:2008 [24]): Mg—base, Nd—from 2.2 to 2.8% by weight, Zr—from 0.4 to 1.0% by weight, Zn—from 0.1 to 0.7% by weight. At the same time, the content of impurities is: Fe—no more than 0.01% by weight, Si—no more than 0.03% by weight, Ni—no more than 0.005% by weight, Al—no more than 0.02% by weight, Cu—no more than 0.03% by weight, Be—no more than 0.001% by weight.

Experimental melts were carried out in an IPM-500 (ProgramTherm, Luoyang, Henan, China) crucible oven, into which preheated bulk materials were loaded and poured into tapping crucibles at 650–730 °C after melting. The tapping crucibles were installed in dispensing furnaces, in which the alloy was refined by chemical composition and refined with Bi-2 flux at 740–760 °C. Next, increasing additions of ligatures containing 999.9 sample Au and Ag were introduced into the melt, then heated; after this, the melt was maintained at 730 °C and samples for mechanical testing were poured into a sandy clay form. For each of the compounds selected for further research, there were provided 5 samples.

The metal was studied after heat treatment in a Bellevue-type thermal mine furnace with a capacity of 112 kW and a productivity of 95 kg/h, as well as in a PAP-4M (ProgramTherm, Luoyang, Henan, China) thermal furnace with a productivity of 50 kg/h, according to the following mode: heating at 540 ± 5 °C, holding for 8 h, cooling in air and aging at 200 ± 5 °C, holding for 15 h, cooling in air.

The quality of the samples was determined using the pulse X-ray method with RAP and MIRA-2D devices. Tensile mechanical properties of the magnesium alloy samples were tested on an INSTRON 2801 (Norwood, MA, USA) universal testing machine. Mechanical properties were determined both on standard samples from magnesium alloys according to DSTU 2824-94 [25], and on similar samples after their aging in artificial blood substitutes—gelofusin (active substances—sodium chloride + gelatin), venofundin (active substances—hydroxyethyl starch + sodium chloride) and physiological solution (aqueous solution of sodium chloride), during 3 periods (measurements were taken after 1, 2 and 3 months) at a temperature of 36 ± 1.0 °C with the usage of the UT-15 ultra-thermostat. When measuring deformation, the rate of deformation was controlled (5 mm/min). The medium was changed once a week; samples in containers remained in a suspended and fixed state.

The macro- and microstructure of the samples after mechanical tests was studied using light microscopy methods with Neophot 32 (Carl Zeiss, Jena, Germany) and OLYMPUS IX 70 (Kiev, Ukraine) devices on pre-polished and etched samples according to DSTU ISO 16220:2008 [24]. For analysis, samples with a diameter of 10 mm and a height of 10 mm were used. Due to the small dimensions of the samples, it was assumed that the cooling rate would not affect the segregation of chemical elements. The chemical composition of structural components of magnesium alloys was determined by electronic microscope—a microanalyzer with the energy dispersive microanalysis (EDS) system “SELMI PEM-106Й”. Quantitative characteristics of intermetallic phases were determined using the linear Rosyval method, which is based on the calculated sums of straight-line segment lengths (intersecting the image of the microstructure) at each phase, with the further distribution of the total length of the segments falling into this phase according to the total length of the straight line.

## 3. Results

**Figure 1:**
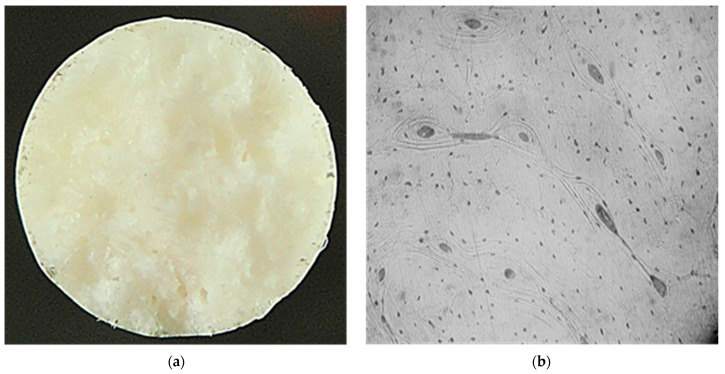
Macro- ((**a**) ×5) and microstructure ((**b**), ×100) of bone tissue samples after mechanical tests.

It has been established that the mechanical properties of the implant material should be as close as possible to the properties of bone tissue and be maintained until the fracture is completely consolidated (up to 3 months) [26,27]. Data analysis of the mechanical properties of human bone tissue shows that they are influenced by a large variety of factors, which causes the properties to vary widely. To determine the structure and level of properties of bone tissue, metallographic and mechanical experiments were performed on standard samples made of bone material. The metallographic studies revealed that the fracture surface of the bone samples has a finely tuberculated appearance, and the presence of „pit” breaks was observed. The nature of the fracture is brittle. The microstructure of the samples consists of osteons, which are blood vessels and nerve fibers surrounded by concentric rings (lamellae), and small osteocytes (Figure 1).

The results of mechanical tests of bone tissue showed that the approximate levels of its physical and mechanical properties are σv ~150 MPa, δ ~3.1%, and E ~20 GPa. At the same time, the material with the mechanical properties closest to bone tissue is magnesium-based alloys (Table 1) [28,29].

Under investigation was the NZ30K alloy of the Mg–Zr–Nd system, which does not contain toxic alloying elements and may be promising for the manufacture of implants. The main disadvantage of this alloy is a decrease in mechanical properties under the influence of biocorrosion. Study of the mechanical properties of the test specimens after aging them in artificial blood substitutes showed that by the third month of aging (average fracture consolidation time), the specimens lost a significant part of their strength and ductility (Table 2) and did not correspond to the level of bone tissue properties.

To improve the mechanical properties of the magnesium alloy under study, the effect of Au and Ag on the morphology and topology of the intermetallic phase that was formed and their relationship with the resulting metal properties was investigated.

Analysis of the macrostructures of the NZ30K alloy samples with 0.05% Au and Ag showed a decrease in distance between the axes of the second order dendrites from 24 to 20–19 μm. With an increase in the content of the studied elements, this value decreased to 18–16 μm, and combined modification with silver and gold in amounts of 0.1% each reduced it to 15 μm.

**Figure 2:**
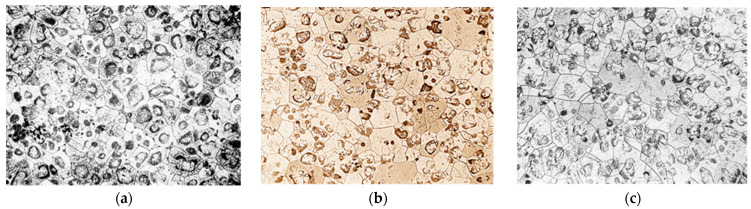
Microstructure of NZ30K alloy (×100): (**a**) initial; (**b**) 0.1% Au; (**c**) 0.1% Ag.

The microstructure of NZ30K alloy was a solid solution, a eutectoid in the form of spherical formations and individual intermetallics. The introduction of silver and gold into the alloy crushed its micrograins as they became additional centers of crystallization. At the same time, silver additives contributed to stronger grain grinding than gold (Figure 2).

**Figure 3:**
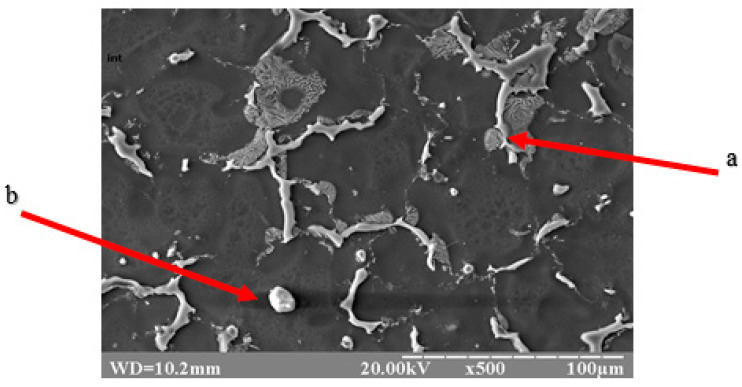
Topology of intermetallics of the NZ30K alloy (lamellar (a) and spherical (b) particles of intermetallic phases).

**Figure 4:**
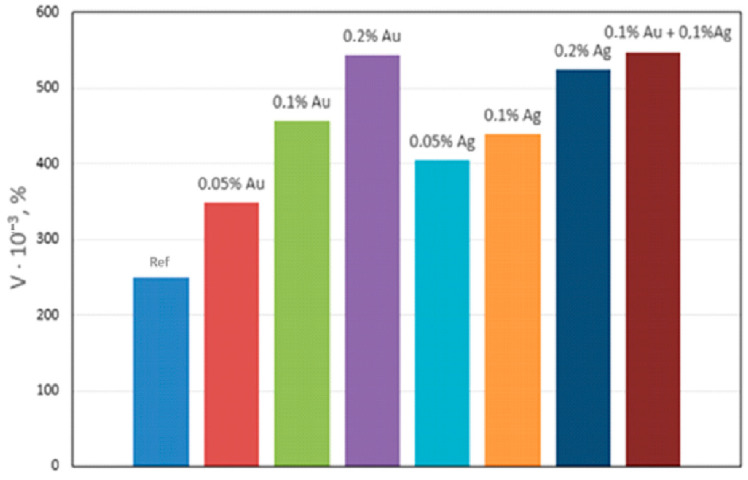
Effect of Ag and Au content on the volume percentage (V) of intermetallics in NZ30K alloy.

Qualitative metallographic analysis showed the presence in the alloy structure of both lamellar and spherical intermetallic phases of complex composition containing noble metals (Figure 3, Table 3). Quantitative metallographic analysis showed that an increase in the content of the studied alloying elements in the alloy increased the volume percentage of the intermetallic phase (Figure 4).

**Figure 5:**
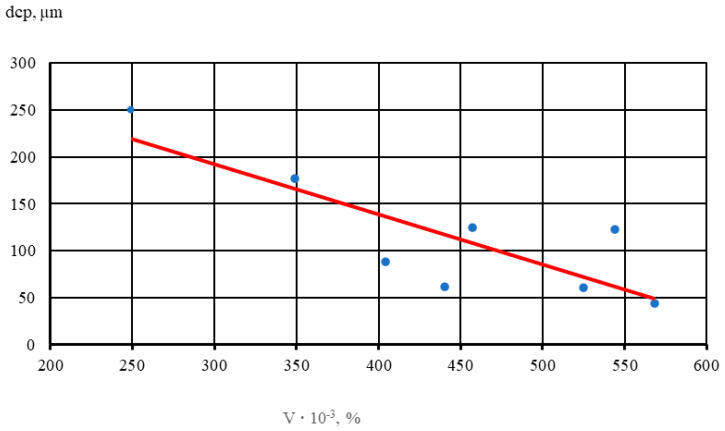
Effect of the volume percentage of intermetallics (V) on micrograin size (dsrp) in the NZ30K alloy.

The results show that with an increase in the volume percentage of intermetallics, there is noticeable grain refinement. Thus, with a 2-fold increase in the volume percentage of intermetallics, the average diameter of the alloy micrograin decreases by half. In Figure 5, dots indicate the average values of five samples of each alloy.

A linear dependence (1) of the influence of the volume percentage of intermetallics with increasing Au and Ag content in NZ30K alloy on the size of the micrograin was established,

(1) $$d_{cp}=y=352.47-0.5344V,\;r=-0.83$$

where:

d_cp_—average micrograin diameter, µm;
V—volume percentage of intermetallics in the alloy, %.

At content of the studied elements in the magnesium alloy in the amount of 0.05–0.1%, the volume percentage of spherical intermetallics increases intensively and that of lamellar ones not significantly (Table 4). With an increase in content of elements in the alloy up to 0.2%, the volume percentage of spherical inclusions located in the middle of the grain and intensively lamellar inclusions does not increase significantly.

Analysis of the distribution of intermetallics by different size groups showed that lamellar intermetallics prevailed in the original NZ30K alloy, most of which were in the size range of 4–15 μm. Spherical intermetallics were mainly represented in the size range of 2.0–7.9 μm. In the magnesium alloy, the alloying elements under study crushed the intermetallic phases, of which the distribution shifted towards smaller groups (to 2.0–11.5 μm for spherical and <2.0–7.9 μm for lamellar). At the same time, an increase in the content of elements in the alloy increased the volume percentage of intermetallics with sizes less than 2 μm and reduced the volume fraction of large intermetallics larger than 11.6 μm.

**Figure 6:**
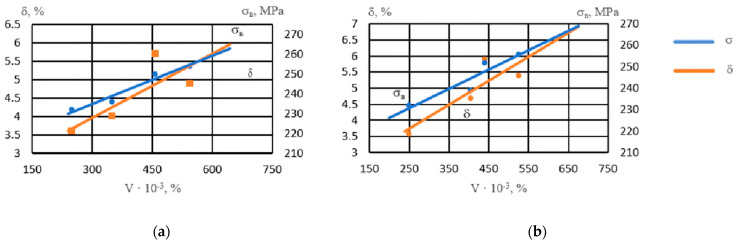
Influence of the volume percentage of intermetallics (V) on the properties of NZ30K alloy: (**a**) Ag; (**b**) Au.

With an increase in the volume percentage of intermetallics, the strength of the alloy increased (Figure 6). Intermetallics located both in the center of the grain and along its boundaries strengthened the alloy. The ductility of the alloy with respect to the number of intermetallics had a nonlinear dependence, reaching maximum values at a noble metal content of 0.05–0.1% due to grain refinement and an increase in the number of spherical intermetallics of smaller size groups.

The resulting dependences are described by these regression equations:

(2) $$\delta \;\mathrm{when}\;\mathrm{adding}\;\mathrm{Ag}:\;y=-3\;\times \;10^{{-05x}^{2}}+0.0278x-1.7793,\;R^{2}=0.80$$

σ_*B*_ when adding Ag: y = 0.081*x* + 210.61, R^2^ = 0.95 (3)

(4) $$\delta \;\mathrm{when}\;\mathrm{adding}\;\mathrm{Au}:\;y=-3\;\times \;10^{{-05x}^{2}}+0.0283x-1.7821,\;R^{2}=0.80$$

σ_*B*_ when adding Au: y = 0.0901*x* + 208.32, R^2^ = 0.85 (5)

In addition, the highest level of mechanical properties was obtained with the combined modification (0.10% Au + 0.10% Ag); namely, with this variant, the strength reached s*_v_* = 262 MPa and the ductility d = 6.2%.

Study of the biodegradation rate of this NZ30K alloy with 0.1% Au and 0.1% Ag (Table 5) showed that after 3 months of exposure to artificial blood substitutes, the developed alloy lost up to 30% of its strength and ductility. At the same time, its mechanical properties exceeded the corresponding properties of bone tissue, which ensured reliable fracture growth with subsequent biodegradation of the implant.

To identify possible negative effects of the experimental alloy and its biodegradation products on bone tissue restoration processes and on the living organism as a whole, additional medical tests were conducted.

## 4. Pre-Clinical and Clinical Trials

Preclinical trials of the experimental alloy samples were conducted at Zaporizhzhia State University of Medicine and Pharmacy, which included research on the effect of metal bioresorption on the process of regenerative osteogenesis, as well as toxicological and bacteriological studies. Clinical trials were conducted at the clinic of JSC “Motor Sich”. Preclinical studies were conducted according to the Order of the Ministry of Education and Science, Youth and Sports of Ukraine No. 249 dated 1 March 2012 on the approval of procedures for conducting experiments on animals by scientific institutions.

Toxicological studies of the effect of resorption products of the experimental magnesium alloy on the body were performed on 20 white outbred male rats weighing 220–270 g. In 14 animals in the experimental group, a fixator made of modified magnesium alloy was implanted in the thigh muscle mass. Six rats that did not undergo surgical intervention (intact group) were used as controls. Rats of both groups were kept in standard vivarium conditions. Changes in animal behavior after implantation of magnesium alloys were studied to assess the overall toxic effect of the implants. Additionally, biochemical studies of metabolic parameters of experimental animals and toxic effects of alloy biodegradation products were performed.

The gradual (during 7 months) metabolism of metal fixtures of a bioreversible magnesium alloy by the body of white male mongrel rats showed no differences in the level of stable nitric oxide metabolites between animals of both groups from day 1 to 6 months after implantation of the alloy. The most significant increases in the index were detected on the 2nd and 14th days and one month after surgery, by 63, 52, and 61%, respectively, indicating the absence of oxidative stress.

Study of the toxic effect of the bioresorption products of the experimental alloy in the experiment on laboratory rats proved an absence of behavioral changes and adverse effects on general condition (no pathological changes in the eyes, fur, or mucous membranes; no changes in body weight), high motor and research activity was maintained, and there was no neurological deficit and no deviations in emotional state. The experimental biochemical study showed a significant increase in the content of all fractions of medium molecular weight peptides in the plasma of white rats after the implantation of the fixator made of experimental magnesium alloy into the femur. The fraction of peptides with a maximum absorbance at 254 nm in the experimental group increased 1.19 times; 272 nm—1.3 times, 280 nm—1.27 times. This indicates a reactive state in the immune system of animals with a small release of biologically active substances into the bloodstream and an absence of endogenous intoxication, in which this indicator increases tenfold. Thus, the bioresorption products of the modified magnesium alloy do not cause toxic effects to body tissues and do not enhance cellular destruction.

In order to determine the effect of magnesium alloy bioresorption on the process of regenerative osteogenesis in the case of fracture, 12 sexually mature rabbits were used. Fractures of the upper third of both femurs were modeled. In the main group, intramedullary osteosynthesis was performed with fixators made of the studied magnesium alloy, and in the control group with rods made of rust-resistant steel 12X18M10T. Control was carried out by conducting an X-ray examination.

As a result of the experimental morphological study, it was proved that implants made of the experimental alloy did not disrupt processes of reparative regeneration of bone tissue, did not suppress processes of vascularization and angiogenesis, and did not affect the proliferative activity of cells involved in bone formation, unlike stainless steel fixtures.

Additionally, the bactericidal activity of the magnesium alloy was studied. For this study, 68 clinical strains belonging to different groups of microorganisms were selected. Magnesium alloy in a liquid medium has high bactericidal activity due to the formation of metal biodegradation products as a result of electrochemical reactions and a shift in the pH of the medium to the alkaline side—from 7.4 to 9.6. Experimental studies have shown that Gram-negative microorganisms of the Enterobacteriaceae family are highly sensitive to magnesium alloy extract, which is confirmed by the cessation of growth of the test strain E.coli ATCC 25922 within 3 days. The growth of *P. aeruginosa* strain ATCC 27853 was inhibited within 72 h. Known pathogens of hospital-acquired surgical infections (*K. pneumoniae*, *E. coli*, *P. mirabilis*, *E. agglomerans*, *E. sakazakii*, *A. baumannii* and *P. aeruginosa*) with multidrug resistance were not resistant to the action of magnesium alloy biodegradation products—their survival time in the presence of alloy degradation products did not exceed 72 h and did not depend on antibiotic susceptibility.

For clinical trials, a 3.5 mm diameter malleolar screw with a short thread made of a bioabsorbable magnesium alloy was chosen as an implant. The results of treatment of 15 patients (12 men and 3 women) with fractures of the internal ossicle were analyzed. The average age of the patients was 50.7 years. Three patients had an isolated fracture of the inner tibia, twelve patients had also broken the inner tibia, and in eight patients there was also damage to the intertibial syndesmosis. These patients underwent osteosynthesis with an extramedullary plate on the external tibia with fixation of the intertibial syndesmosis with a locked screw according to a typical technique.

The results of clinical testing of the magnesium-based malleolar screw in osteosynthesis of fracture of the inner tibia showed that bioresorption of the implant is not accompanied by clinical manifestations and complications, and does not affect the timing of soft tissue healing and the formation of bone regeneration in the fracture zone.

## 5. Discussion

The role of the morphology and topology of strengthening intermetallic phases in the formation of the structure and the improvement of mechanical properties of a magnesium-based alloy modified with noble metals was established. It was shown that the properties of the magnesium alloy are affected not only by the amount of intermetallic phase that is released but also by its morphology and topology. Lamellar intermetallics up to 8.0 μm in size have a positive effect on the properties of the alloy, while spherical intermetallics have a positive effect only up to a size of 11.6 μm. Accordingly, the spherical form of intermetallics is preferable for improving the properties of the alloy. It was found that spherical intermetallics of smaller size groups have a greater effect on alloy hardening. An increase in alloy plasticity was observed only when the studied elements were introduced in amounts of 0.05–0.1%, with the proportion of spherical intermetallics increasing from grain to grain. With a further increase in the content of elements in the alloy (up to 0.2%), simultaneously with the grinding of the micrograins, the volume percentage of intermetallics increased, which led to metal embrittlement and a decrease in relative elongation. Gold and silver in amounts of 0.05–0.1% contributed to improvement in the entire set of alloy properties. Their further increase (up to 0.2%) contributed to additional strengthening and a certain decrease in ductility. The optimal content of noble metals in the magnesium alloy (~0.1%) ensures increased levels of its mechanical properties over 3 months of exposure to artificial blood substitutes, which corresponds with the properties of bone tissue.

Preclinical studies showed that biodegradation products of implants made of this modified magnesium-based alloy do not affect the regeneration of bone tissue in laboratory rabbits. Based on biochemical studies, it was determined that the biosorption products of the developed alloy do not affect the body in laboratory rats (no signs of intoxication were found) and do not lead to behavioral changes, i.e., have no neurotoxic effect.

The bactericidal effect of the developed alloy bioresorption products on cultures of reference test strains of *S. aureus*, *E. coli* and *P. aeruginosa*, clinical strains of the Enterobacteriaceae family, and non-fermenting Gram-negative microorganisms *A. baumannii* and *P. aeruginosa*, *staphylococci* and *enterococci* were studied.

The results of clinical testing showed that the use of a molecular screw made of the developed magnesium-based alloy does not affect the healing time of medial malleolus fractures and does not cause complications of the wound process. Clinical testing of structures made of the developed magnesium-based alloy revealed high efficiency in cases of bone fractures due to a positive effect on osteoreparation, the absence of toxicological reactions, and high biocompatibility. The advantage of the developed structures is their bioresorption with simultaneous replacement by bone tissue. The use of biosoluble magnesium alloy implants for osteosynthesis of fractures allows for a reduction in the period of temporary disability, due to the absence of a second surgery to remove the metal implant, and improves the quality of treatment and life of patients.

## 6. Conclusions

1. The positive effect of silver and gold on the structure and mechanical properties of NZ30K magnesium alloy was established. Separate and combined contents of Ag and Au in the alloy in amounts from 0.05 to 0.2 wt.% contributed to a significant reduction in the distance between the dendrites’ axes of the second order and the size of the micrograins.
2. Analyses showed that modification of magnesium alloy with silver and gold in amounts of 0.05 to 0.2 wt.% increased the volume percentage of intermetallics by ~1.5 times, shifting them towards smaller size groups with the formation of spherical intermetallics of complex composition located in the center of the grain that serve as additional crystallization centers.
3. It was found that the combined modification (0.1% Ag + 0.1% Au) of the magnesium alloy reduced the size of its structural components and contributed to a significant increase in its mechanical properties after aging in artificial blood substitutes for three months. The results of preclinical tests showed that the developed alloy has no toxic or carcinogenic effects on a living organism and has a positive effect on osteogenesis and reparative processes.
4. Clinical testing of magnesium-based implants for osteosynthesis of internal bone fracture showed that implant bioresorption is not followed by clinical manifestations or complications, and does not affect the timing of soft tissue healing and bone regeneration in the fracture area. Based on experimental and clinical study, the usage of implants made of magnesium-based biorefining alloy for bone osteosynthesis was substantiated, as they do not require an additional surgery for their removal, do not affect osteogenesis, and do not cause local or systemic reactions.

## Figures and Tables

**Table 1:** Properties of various metals and alloys used for the manufacture of implants [28,29,30].

Material	Ultimate Tensile Strength σ*_B_*, MPa	Relative Elongation in Tensile Test δ, %	Young’s Modulus E, GPa
Alloys based on magnesium	200–280	2–7	44–45
Bone tissue	Up to 150	Up to 3.1	Up to 20

**Table 2:** Mechanical properties of NZ30K alloy after aging in artificial blood substitutes.

Environment	Original		After 1 Month		After 2 Months		After 3 Months	
	σ*_B_*, MPa	δ, %	σ_B_, MPa	δ, %	σ*_B_*, MPa	δ, %	σ*_B_*, MPa	δ, %
Gelofusin	233.2	3.3	179	2.5	142	1.8	113	1.1
Venofundin	234.1	3.4	163	2.3	136	1.6	98	0.8
Physiological solution	233.5	3.3	186	2.7	151	2.1	122	0.9

**Table 3:** Chemical composition of intermetallics in NZ30K alloy doped with noble metals.

Alloy Doping Option	Content of Elements in Intermetallics, % by Mass.					
	Modifier	Nd	Zr	Fe	Mn	Mg
Au	1.77	3.04	2.45	0.25	0.19	92.30
Ag	1.55	2.76	0.69	0.09	0.49	94.42

**Table 4:** Volume percentage of intermetallics (V) and their distribution by size groups in NZ30K alloys with Au and Ag.

Modifier	Contents, %	Distribution of Intermetallics (V·10^−3^, %) by Size Groups, μm						
		<2	2–3.9	4–7.9	8–11.5	11.6–15	15.1–19	Total
standard		7/0 *	19/53 *	37/29 *	31/11 *	31/12 *	19/0 *	144/105 *
Au	0.05	40/1	82/75	21/43	11/25	31/0	19/1	204/145
	0.1	65/2	91/155	43/43	11/19	19/1	7/1	236/221
	0.2	76/79	160/129	42/25	6/7	18/1	0/1	302/242
Ag	0.05	47/1	48/110	31/43	25/37	35/7	19/1	205/199
	0.1	91/19	29/116	47/44	19/32	19/7	16/1	221/219
	0.2	147/62	25/116	73/34	19/26	11/7	5/0	280/245
0.1% Au + 0.1% Ag		143/112	111/119	69/71	9/14	0/0	0/0	232/316

* The numerator is the volume percentage of lamellar intermetallics, and the denominator is the volume percentage of spherical intermetallics.

**Table 5:** Mechanical properties of the NZ30K alloy with 0.1% Au and 0.1% Ag aged in artificial blood substitutes.

Environment	Original (Not Aged)		After 1 Month		After 2 Months		After 3 Months	
	σ*_B_*, MPa	δ, %	σ*_B_*, MPa	δ, %	σ*_B_*, MPa	δ, %	σ*_B_*, MPa	δ, %
Gelofusin	271	4.4	246	4.1	220	3.7	188	3.3
Venofundin	266	4.8	238	4.3	212	3.7	171	3.2
Physiological solution	270	4.6	259	4.4	241	4.2	218	3.8

## Data Availability

The original contributions presented in the study are included in the article, further inquiries can be directed to the corresponding authors.
